# Vehicle-Assisted UAV Delivery Scheme Considering Energy Consumption for Instant Delivery

**DOI:** 10.3390/s22052045

**Published:** 2022-03-05

**Authors:** Xudong Deng, Mingke Guan, Yunfeng Ma, Xijie Yang, Ting Xiang

**Affiliations:** Evergrande School of Management, Wuhan University of Science and Technology, Wuhan 430065, China; guanmingke@163.com (M.G.); mayunfeng@wust.edu.cn (Y.M.); yangxj97@wust.edu.cn (X.Y.); xiangting0912@163.com (T.X.)

**Keywords:** unmanned aerial vehicle, vehicle routing problem, instant delivery

## Abstract

Unmanned aerial vehicles (UAVs) are increasingly used in instant delivery scenarios. The combined delivery of vehicles and UAVs has many advantages compared to their respective separate delivery, which can greatly improve delivery efficiency. Although a few studies in the literature have explored the issue of vehicle-assisted UAV delivery, we did not find any studies on the scenario of an UAV serving several customers. This study aims to design a new vehicle-assisted UAV delivery solution that allows UAVs to serve multiple customers in a single take-off and takes energy consumption into account. A multi-UAV task allocation model and a vehicle path planning model were established to determine the task allocation of the UAVs as well as the path of UAVs and the vehicle, respectively. The model also considered the impact of changing the payload of the UAV on energy consumption, bringing the results closer to reality. Finally, a hybrid heuristic algorithm based on an improved *K*-means algorithm and ant colony optimization (ACO) was proposed to solve the problem, and the effectiveness of the scheme was proven by multi-scale experimental instances and comparative experiments.

## 1. Introduction

In recent years, China’s logistics industry has developed rapidly, giving rise to more subdivision tracks. As an emerging logistics service, instant delivery has developed into an industry with an output value of nearly 100 billion dollars [1] in just a few years and the scope of services has also changed from delivering catering to delivering everything. Data show that in 2015, the number of instant-delivery users in China was 171 million, and in 2020, it reached 506 million [2], with an average annual growth rate of about 24%. With the deepening development of the industry, more traditional express companies and various Internet companies have entered the game, and competition in the field of instant delivery is becoming increasingly fierce. Whoever can provide faster delivery and better service will be able to break through in the instant-delivery market. As an emerging technology, unmanned aerial vehicles (UAVs) have the advantages of fast speed, low cost, and are not affected by urban congestion and terrain limitation [3]; therefore, UAVs are being gradually employed in delivery.

In December 2013, Amazon took the lead in launching an UAV delivery package called Prime Air [4], which delivered packages under 2.5 kg to customers in less than 30 min. Since 2015, JD.com, SF Express, Meituan, Eleme, and other companies in China have continuously obtained UAV logistics pilot and operation qualifications and launched corresponding UAV products and routes. UAVs are gradually being used in parcel delivery, but UAVs have the shortcomings of limited range and insufficient load, and the limitation of independent delivery is relatively large. If UAVs are combined with ground vehicles, coordinated delivery can overcome these shortcomings and improve delivery efficiency. Therefore, how to design an efficient joint delivery scheme of vehicles and UAVs has become an important issue.

The current research on UAV delivery is divided into three categories: (1) UAV independent delivery, whereby the UAV completes the delivery task independently without any vehicle support; (2) the UAVs and the vehicles are distributed in cooperation, and the two are distributed independently of each other without interaction; and (3) joint delivery between UAVs and vehicles, that is, UAVs and vehicles cooperate and participate in the entire delivery process. In this study, we focused on the third category of problems. This type of problem can be subdivided into many directions, for example, Mathew et al. [5] conducted a study where one vehicle carried one UAV; Murray et al. [6], where one vehicle carried multiple UAVs; and GU et al. [7], where multiple vehicles carried multiple UAVs.

However, it is worth noting that numerous existing studies have tended to assume that UAVs serve only one customer in a single take-off, which does not match the current development of UAV technology. For example, the FreeFly Alta 8 is capable of carrying packages of up to 18 kg [8]. DJI also produces UAVs capable of carrying multiple packages such as the DJI Spreading Wings S900, which is capable of carrying 8.2 kg of packages [9], while the vast majority of packages delivered by Amazon (86%) were below 2.3 kg [4]. Therefore, conducting research on the new scenario of UAVs serving multiple customers with a single take-off will further improve delivery efficiency, which is necessary for instant delivery.

However, the change in the payload of the UAV will greatly affect its flight distance [10]. If the influence of the change of the payload of the UAV on energy consumption can be considered in the model and algorithm, the solution will be closer to reality.

To this end, the focus of this study was to design a new vehicle-assisted UAV delivery solution that allows UAVs to serve multiple customers in a single take-off and takes energy consumption into account. Our goal was to minimize the total service time including the total UAV delivery time for each cluster and vehicle operation time. Our work makes the following three contributions: (1) We established models to solve the new problem that a vehicle is equipped with multiple UAVs and the UAV serves multiple customers in a single take-off; (2) the model considered the impact of changes in the payload of the UAV on energy consumption, making the results closer to reality; and (3) a hybrid heuristic algorithm was proposed to solve the problem, and the effectiveness of the scheme was proven through multi-scale examples and comparative experiments.

The remainder of this paper is organized as follows. Section 2 reviews the relevant literature. Section 3 describes the problem and explains the model. Section 4 presents and explains the heuristic algorithms for running the models. Section 5 uses different examples to verify the validity of the model and algorithms. Conclusions and future work are discussed in Section 6.

## 2. Literature Review

The vehicle routing problem (VRP) is the basis for the development of research on the joint delivery of vehicles and UAVs. It was first proposed by Dantzig and Ramser (1959) [11] to solve the practical problems of gasoline transportation routes. After more than 60 years of development, the VRP problem research has expanded to VRP with loading restrictions (Lijun Wei et al.) [12], VRP with multiple vehicles (Zheng Wang et al.) [13], VRP with time windows (Vitória et al.) [14], VRP with simultaneous pickup and delivery (The Jin Ai et al.) [15], and so on. Among them, the time dependent vehicle routing problems (TDVRP) (Malandraki et al.) [16] inspired the instant-delivery operation with the goal of minimizing the vehicle travel time. However, these studies did not consider UAVs as an alternative delivery tool.

Some studies have considered scenarios where UAVs are independently delivered. Chitta and Jain [17] pointed out that UAVs have great application value in the logistics field, especially in improving delivery speed. Sundar and Rathinam [18] considered a single UAV route problem and minimized the total fuel required for UAVs to access all targets.

The above studies set the endurance of the UAV to a fixed value, but based on the actual situation, the impact of the payload on the energy consumption of the UAV during the flight cannot be ignored. Song B. D. et al. [19] discussed the influence of cargo weight on flight capability. Dorling et al. [20] proposed two variants of the VRP problem for UAV delivery using a linear approximation function to calculate the linear change of energy consumption with the effective load and battery weight. Mariga et al. [21] proposed a method to measure the UAV battery discharge curve using the LabVIEW interface and a low-cost acquisition system to better estimate the UAV’s endurance. Jung et al. [22] used the concept of state of charge (SOC) estimation based on the extended Kalman filter (EKF) and complementary filter (CF) and then calculated the possible flight time by using the slope of the SOC graph during hovering flight mode. Maryam et al. [23] proposed the battery consumption rate (BCR), which demonstrated the impact of the UAV battery consumption on the design of the UAV package–delivery system. However, research on these issues is limited to the independent delivery of UAVs, and vehicles have not been considered.

Due to limited battery life and load capacity, the disadvantages of independent delivery of UAVs are obvious. If vehicles and UAVs are used together for distribution, it will greatly improve the delivery efficiency. In recent years, many related studies have appeared.

In 2015, Murray et al. [24] first proposed adding UAVs to the research of the traveling salesman problem (TSP), called the flying sidekick traveling salesman problem (FSTSP), where the vehicle is equipped with a drone to complete package delivery, and it is stipulated that the UAV can only serve one customer in a single journey. They set up a mixed integer linear programming (MILP) model and designed a heuristic algorithm to solve the problem to minimize the completion time. Mathew et al. [5] described this problem as the heterogeneous delivery problem (HDP) and discussed that one truck supported delivery by one UAV, the vehicle provided long-distance transportation and support, and the UAV completed the last part of each delivery to reduce the overall delivery time and fuel consumption. Dayarian et al. [25] demonstrated the potential benefits of using UAVs to resupply vehicles, both increasing the number of service orders and reducing the service time. Sara et al. [26] and Poikonen et al. [27] proposed precise algorithms to solve the problem of a vehicle carrying multiple UAVs, but are not suitable for large-scale situations. Ermağan et al. [28] and Wu et al. [29] proposed intelligent heuristics to solve this problem, suitable for large-scale situations. Chang et al. [30] considered the delivery problem of a single vehicle carrying multiple UAVs, still assuming that the UAVs could only serve one customer in a single trip, clustering the customer points using the K-means algorithm, and assuming that the vehicle carried enough UAVs, all UAVs could finish serving the customers in each cluster in a single take-off. In addition, a shift-weights process was added to the selection of vehicle stops to shorten the total delivery time. Gu et al. [7] considered the scenario of multiple vehicles carrying multiple drones and determined the location of the vehicle stopping point, the assignment of customers, the assignment of UAVs, and the route of the vehicles by building two models and solved the problem with two improved ant colony optimizations (ACOs). Bakir et al. [31] put the problem as a mixed-integer linear program on a time–space network and presented an efficient optimization algorithm based on a dynamic discretization discovery approach. The above research often assumed that the UAVs served one customer per take-off and did not consider the energy consumption of the UAVs.

In summary, most researchers have assumed that UAVs only served one customer at a single take-off, and the endurance of UAVs was often only set at a fixed value, which deviates greatly from the actual situation. Therefore, this study focused on a new scenario in which multiple packages can be delivered on the flight path of an UAV and considers the impact of changes in the payload on the energy consumption of the UAV. Given this, our study established a UAV energy consumption model to constrain the maximum flight distance of UAVs under different load conditions and established an UAV task allocation model and a vehicle path planning model to determine the UAVs’ task allocation and path as well as the vehicle running path and designed a hybrid heuristic algorithm to solve them, and finally verified the feasibility of the algorithm with calculation examples.

## 3. Problem Description

**Figure 1:**
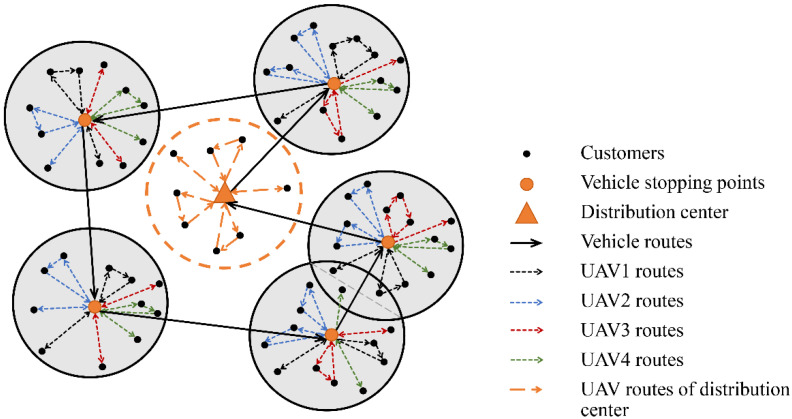
Description of vehicle-assisted UAV delivery.

The problem that forms the basis for this study can be described as follows: a vehicle carrying multiple UAVs starts from the distribution center, traverses the vehicle stopping points in each customer cluster in turn, and then returns to the distribution center. As shown in Figure 1, when the vehicle arrives at the stopping point, multiple UAVs load the packages and take-off at the same time, deliver the packages and return to the vehicle under the constraints of the energy consumption model, and repeat the delivery until all customers in the cluster have been serviced. The vehicle drives to the next stopping point. In this situation, the vehicle is only used as a mobile warehouse and UAV charging station, and customers near the distribution center are directly delivered by UAVs. Our goal was to minimize the total service time including the UAVs’ total delivery time for each cluster and vehicle operation time.

### 3.1. Problem Assumptions

(1) Each UAV is homogeneous;
(2) Negligible time for take-off, landing, and battery replacement for each UAV;
(3) The UAV has a fixed service time for each customer;
(4) The UAV can serve multiple customers per take-off within its carrying capacity;
(5) The energy consumption of the UAV when taking off and landing is the same as when traveling; and
(6) The distribution center has enough UAVs to meet the customers who are directly delivered by UAVs near the distribution center, and the service time is much shorter than the service time of the remaining customer points.

### 3.2. Problem Models

The notations that appear in the model are given in Table 1.

#### 3.2.1. UAV Energy-Consumption Model

**Figure 2:**
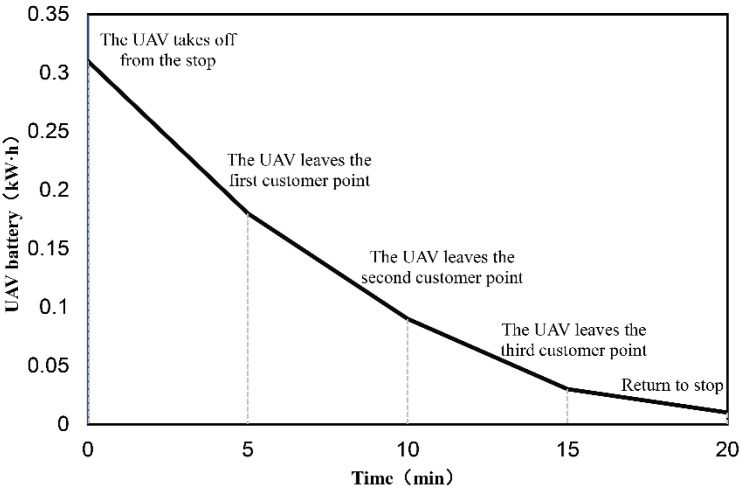
Energy-consumption process of an UAV carrying three packages for delivery.

The maximum flight distance of an UAV is closely related to its energy consumption, which depends on its own weight and the weight of the packages it carries. The delivery of UAV should be completed under the limitation of battery energy. If the flight time or flight distance of UAV is simply limited without considering the energy consumption, the UAV may not be able to complete the delivery task due to insufficient battery power. Conversely, the battery is not fully utilized, and more UAVs need to be dispatched for delivery, which makes the cost increase. Therefore, it is necessary to consider energy consumption during UAV delivery. In the case of taking off to serve multiple customers at one time, the UAV will load all the corresponding packages in order, then serve the customers sequentially according to the designated route and unload the packages one by one. Once the packages are dropped off at the customer points, the load will gradually be reduced, as will the energy consumption. Since the energy-consumption rate can be considered to vary linearly with the payload [10], this process was fitted to better illustrate the process. Figure 2 shows the energy-consumption process of an UAV carrying three packages in a sub-path for delivery. As packages are delivered to customers in turn, the payload of the UAV gradually decreases, as does the rate of energy consumption.

We assumed that the UAV maintained the maximum power during flight, and combined with the discussion by D’Andrea et al. [10] on the energy consumption of UAVs, we established an UAV energy-consumption model to estimate the energy consumption of UAVs when the effective load and power vary, and then calculated the real travelable distance of UAV. Therefore, the flight speed and flight time from point i to j can be estimated as follows:

(1) $$v_{ij}=\frac{370\eta \gamma \left(P-e\right)}{w_{d}+G_{i}}$$

(2) $$t_{ij}=\frac{d_{ij}\left(w_{d}+G_{i}\right)}{370\eta \gamma \left(P-e\right)}$$

where η is the conversion efficiency of the engine; γ is the lift ratio; e is the energy loss of the UAV battery; $w_{d}$ is the weight of the UAV; $G_{i}$ is the payload when the UAV leaves i; $d_{ij}$ is the distance of the UAV when it leaves i and arrives at j; and $t_{ij}$ is the flight time of the UAV when it leaves i and arrives at j. Therefore, the relationship between UAV payload and energy consumption can be expressed as:

(3) $$E_{ij}=Pt_{ij}$$

where $E_{ij}$ is the energy consumed when the UAV leaves i  and arrives at j. When the UAV model is determined, the unknown parameters in Equations (1)–(3) can be clarified. Based on the above UAV energy-consumption model, we can calculate the total energy consumed by the UAV for each UAV delivery line and thus determine whether it exceeds the battery capacity. It should be noted that our calculation ignores the influence of weather condition, and how the energy consumption is affected by the weather condition may require additional estimates under different weather conditions.

#### 3.2.2. UAV Task Assignment Model

To solve the problem of task allocation between customers and UAVs in the cluster and the confirmation of UAV delivery routes, an UAV task-assignment model was established. The goal of this model is to minimize the waiting time of the vehicle at the stopping point, that is, to minimize the longest UAV service time in the cluster. In particular, the model is based on a single cluster and applies to all customer clusters. The specific formula of the model is as follows:

(4) $$minT^{\prime }$$

(5) $$T^{\prime }=\underset{U}{max}\{T_{U}\},\forall u\in U$$

(6) $$T_{u}=\sum_{r\in R}\sum_{i\in C}\sum_{j\in C}t_{ij}x_{ur}x_{rij}+\sum_{r\in R}\left(\sum_{i\in C}x_{ur}x_{rij}-1\right)t_{s},\forall u\in U$$

(7) $$\sum_{i\in C}\sum_{j\in C}x_{ur}x_{rij}E_{ij}\leq E_{max},\forall u\in U,\forall r\in R$$

(8) $$\sum_{i\in C}x_{ur}x_{rij}w_{i}\leq W_{H},\;\forall u\in U,\forall r\in R,\forall j\in C$$

(9) $$\sum_{u\in U}\sum_{r\in R}x_{ur}x_{rij}=1,\;\forall i,j\in C$$

(10) $$\sum_{u\in U}\sum_{r\in R}x_{ur}x_{rij}=1,\forall i,j\in C$$

(11) $$d_{0j}\leq D_{max},\forall j\in C$$

(12) $$\sum_{j\in C}x_{ur}x_{r0j}=\sum_{i\in C}x_{ur}x_{ri0}=1,\;\forall u\in U,\forall r\in R$$

(13) $$x_{ur}\in \left\{0,1\right\},\forall u\in U,\forall r\in R$$

(14) $$x_{rij}\in \left\{0,1\right\},\forall r\in R,\forall i,j\in C$$

Objective Function (4) minimizes the waiting time of the vehicle at the stopping point. Constraint (5) indicates that the waiting time of the vehicle at the stopping point is the longest UAV service time in the cluster. Constraint (6) indicates that the UAVs’ service time includes the UAVs’ in-transit operation time and the stay time at the customer points where $t_{s}$ is the service time of a single customer. Constraint (7) is the energy consumption constraint of the UAV line where $E_{max}$ is the total energy of the UAV. Constraint (8) is the load constraint of the UAV line. Constraint (9) ensures that each customer is served by only one UAV. Constraint (10) ensures that all customers must be served once. Constraint (11) ensures that all customers are within the scope of the UAV service. Constraint (12) indicates that the UAV must return to the stopping point after starting from the stopping point. Constraints (13) and (14) define the decision variables.

#### 3.2.3. Vehicle-Path Planning Model

The vehicle-path planning model takes the minimum vehicle running time as the objective function to calculate the vehicle travel path. The specific formula of the model is as follows:

(15) $$minT_{travel}$$

(16) $$T_{travel}=\frac{\sum_{i\in K}\sum_{j\in K}k_{ij}p_{ij}}{v_{truck}}$$

(17) $$\sum_{i\in K}k_{ij}=1,\forall j\in K\;$$

(18) $$\sum_{i\in K}\sum_{j\in K}k_{ij}=k$$

(19) $$\sum_{i\in K}k_{0i}=\sum_{i\in K}k_{i0}=1$$

(20) $$\sum_{i\in C}w_{i}\leq W$$

(21) $$\sum_{i\in N}\sum_{j\in N}k_{ij}\leq \left|N\right|-1,\forall N\in K,N\neq \emptyset$$

(22) $$k_{ij}\;\in \left\{0,1\right\},\forall i,j\in K$$

Objective Function (15) minimizes the running time of the vehicle on the way. Constraint (16) indicates the calculation process of the vehicle running time, where $p_{ij}$ is the distance between the vehicle stopping points i and j. Constraints (17) and (18) ensure that all vehicle stopping points are passed and passed only once. Constraint (19) ensures that the vehicle leaves the distribution center and eventually returns to the distribution center. Constraint (20) ensures that the transportation route meets the maximum load of vehicles, where $w_{i}$ is the weight of the package that should be delivered to i. Constraint (21) avoids subloops. Constraint (22) defines the decision variables.

When the waiting time of the vehicle at each stop and the running time of vehicles in transit are calculated, the minimum total service time can be obtained, as shown in Equation (23).

(23) $$T=\sum_{s\in S}T_{s}^{\prime }+T_{travel}$$

## 4. Algorithm Design

In this section, we propose a hybrid heuristic algorithm to deal with the preceding formula and introduce the algorithm flow in detail.

During the process of solving the vehicle-assisted UAV delivery problem, the following decisions must be determined. This includes the selection of customer clusters and vehicle stopping points, the assignment of UAV tasks, and the determination of delivery routes and vehicle operating routes. We determined several sets of feasible customer clusters and vehicle stopping points by improving the K-means algorithm and combined it with the center-of-gravity method. Then, through the improved ACO, the UAV, and vehicle routes are solved and optimized. Due to the existence of multiple sets of feasible customer clusters and vehicle stopping points, the above process produces multiple sets of vehicle and UAV operation schemes. Finally, the goal was to minimize the total service time to obtain the optimal vehicle and UAV operation scheme.

**Figure 3:**
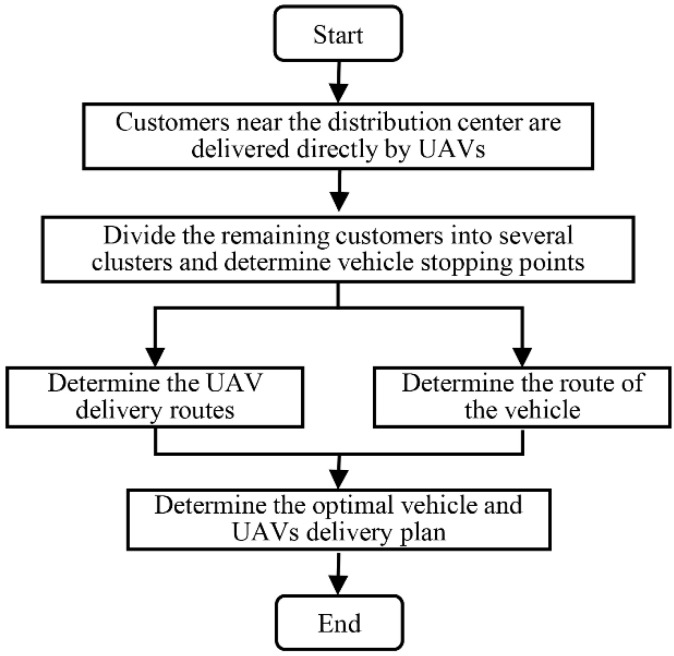
Process for solving vehicle-assisted UAV delivery problems.

The overall solution process is shown in Figure 3, which is divided into the following five steps.

Step 1: UAV delivery has faster delivery speed and lower delivery costs. Customers who meet the UAVs’ service capabilities near the distribution center will be delivered directly by the UAVs, which will further shorten the total service time. With the distribution center as the center of a circle, the maximum service distance of the UAV under full load is the radius of one scan. Customers within the range will be delivered to directly by the UAVs from the distribution center, and customers outside the range will proceed to the next step.

Step 2: The division of customer clusters and the determination of vehicle stopping points. We can divide the remaining customers into the number of clusters and use the center-of-gravity method to determine vehicle stopping points. After the vehicle arrives at the stop, the UAVs take off for delivery and rush to the next stop after the service is completed. Since different cluster divisions will affect the final total service time, the improved K-means algorithm is used to divide multiple sets of feasible customer clusters and carry them into the subsequent steps.

Step 3: Determine the UAVs’ delivery routes. By minimizing the delivery time of the UAV with the longest flight time among all UAVs in each cluster, the total delivery time of UAVs can be minimized. At the same time, each take-off of the UAV is constrained by the energy consumption model and the effective load.

Step 4: Plan the route of the vehicle. After determining multiple sets of feasible vehicle stopping points, the ACO is used to obtain the shortest path of each group of vehicles;

Step 5: Taking the minimum total service time as the goal, we can compare the total time of each group of feasible solutions to determine the optimal vehicle and UAV delivery plan. Among them, the total service time includes the sum of the vehicle’s stay time at each stop and the transit time.

### 4.1. Determination of Customer Clusters and Vehicle Stopping Points

**Figure 4:**
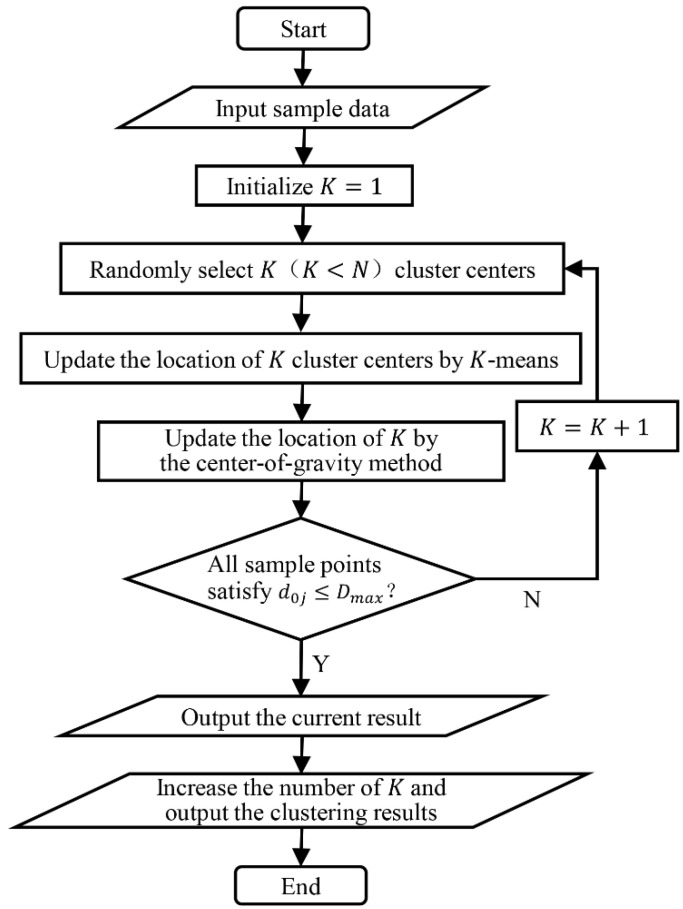
Improved K-means process.

In this study, the improved K-means algorithm was used to divide the remaining customers into several customer clusters, and the center-of-gravity method was used to determine the vehicle stopping point of each cluster. The traditional K-means algorithm [32] needs to specify the initial K cluster centers and then iterate continuously, according to the Euclidean distance between the samples to make the distance between the samples in each cluster as close as possible to achieve the clustering effect. However, for the actual scenario of joint vehicle and UAV delivery, it is difficult to determine the initial K value. Therefore, this study improved the K-means algorithm and combined it with the center-of-gravity method to determine customer clusters and vehicle stopping points. The algorithm flow is shown in Figure 4.

Specific steps are as follows:

Step 1: Initialization. Set the number of samples N  as the maximum number of clusters, and the initial number of clusters K starts from 1.

Step 2: Randomly select K samples from all samples as cluster centers.

Step 3: Assign each sample to the nearest cluster through K-means iteration and update the position of the cluster center until the position of the cluster center can no longer be optimized.

Step 4: Use the center-of-gravity method to optimize the positions of K cluster centers. The center-of-gravity method is a common method in logistics facilities. The center of gravity of a logistics system is often the best setting point for logistics nodes. According to the distance between the sample point and the cluster center and the demand quantity, the distance coefficient and demand weight can be obtained, and the position of the cluster center is updated as the position of the vehicle stopping point.

Step 5: Determine whether all sample points in each cluster are within the service range of the UAV. That is, whether Constraint (11) is satisfied. If it is not satisfied, make K+1 and return to Step 2; if satisfied, continue to run down.

Step 6: Save the current feasible solution.

Step 7: Continue to increase the number of K until the final total service time curve begins to increase and save the result.

In the above process, the division of clusters determines each customer cluster, and the calculation of the cluster center position determines the vehicle stopping point. Among them, Steps 6 and 7 will generate multiple sets of feasible customer cluster division plans, and these plans are substituted into the subsequent steps to find the overall optimal plan.

### 4.2. Determination of the UAVs’ Delivery Routes

After each customer cluster is identified, the delivery routes of the UAVs within the cluster can be calculated. We propose an improved ACO to determine the delivery routes of UAVs. The algorithm is an improvement to the standard ACO [33], and its core is the candidate list, visibility, and pheromone concentration. In this problem, the ant selects the next point j to be visited at the point i through the candidate list. The candidate list is all of the undelivered customer points in the cluster, and Constraints (7) and (8) must be satisfied during the selection.

The visibility calculation of ants is expressed Equation (24).

(24) $$\frac{1}{d_{ij}}$$

**Figure 5:**
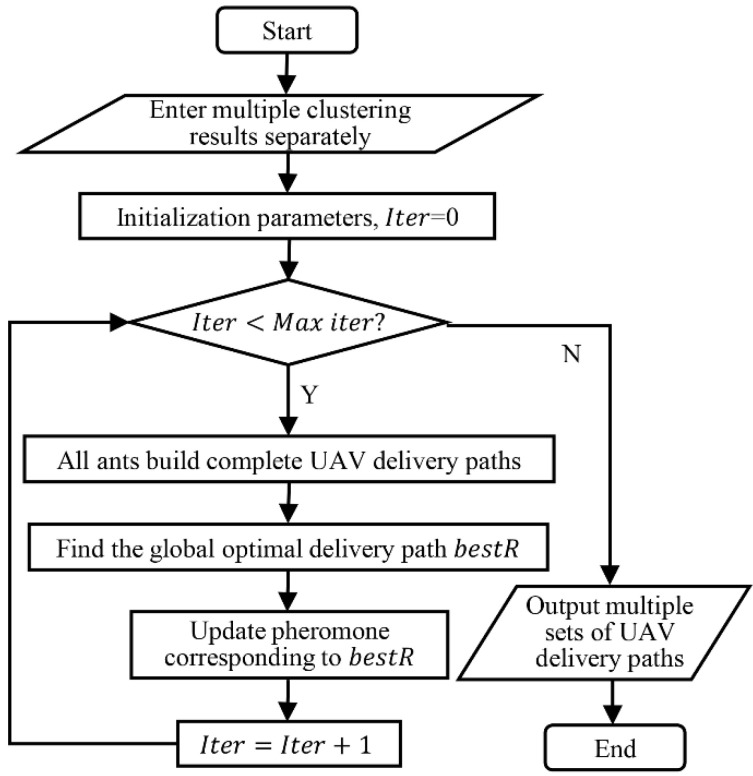
Improved ACO process.

Higher visibility means that the distance between customer points is smaller, and the probability of being selected is higher. After ants pass the customer point, they will leave behind pheromones, and the pheromone concentration τ will influence the selection of subsequent ants. The updated method of pheromone concentration for each iteration is shown in Equations (25) and (26).

(25) $$\tau_{new}=\left(1-\rho \right)\tau_{old}+\Delta \tau$$

(26) $$\Delta \tau =\frac{Q}{\sum_{s\in S}T_{s}^{\prime }}$$

where ρ is the pheromone volatilization coefficient; Δτ is the pheromone increment; and Q is a constant indicating the pheromone intensity. If there are m ants, m kinds of UAV delivery paths will eventually be generated. To make the results converge to the minimum total service time faster, we only updated the pheromone of the shortest UAV service time scheme in each iteration. The specific algorithm flow is shown in Figure 5.

### 4.3. Determining the Route of the Vehicle

When multiple sets of feasible vehicle stopping points are generated, the classic ACO is called to solve them, and multiple sets of vehicle driving routes are obtained. In this way, multiple sets of feasible vehicle and UAV delivery plans will be obtained, with the goal of minimizing the total service time. By comparing the total service time of each plan, the optimal vehicle and UAV delivery plan can finally be determined.

## 5. Simulation and Results

In this section, we present the results of all numerical simulations, and use a case study as an example to illustrate our simulation process.

### 5.1. Simulation Process

Referring to the simulation results of Gu et al. [7] on the number of immediate delivery orders in Shanghai, we randomly generated 15 test examples. The size of the examples ranged from 20 to 250 including large, medium, and small scales, with case numbers from C1 to C15. The calculation examples were randomly and evenly distributed within a radius of 10 km. We solved the examples through MATLAB and CPLEX 12.8. The software runs on a Windows 10 operating system, with Ryzen 9-5000HS 3.30GHz CPU, 16GB memory. In this simulation, it was assumed that a vehicle was equipped with four UAVs for delivery. The parameters used in the simulation are shown in Table 2.

**Figure 6:**
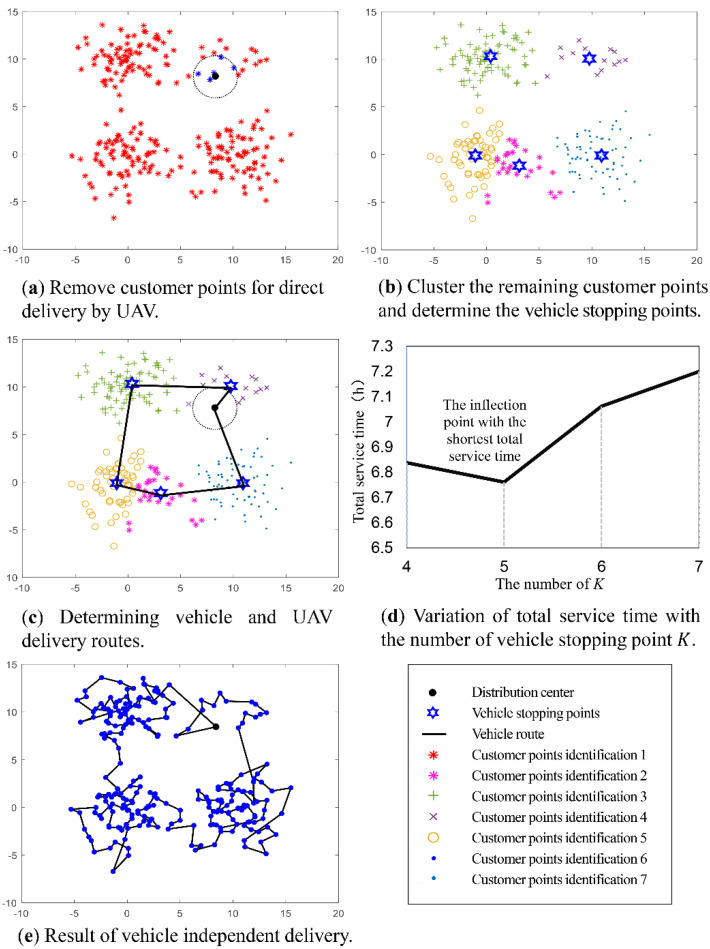
Illustration of the example of C1.

To better demonstrate our simulation process, we used the large-scale case C1 as an example to show the realization process of the vehicle-assisted UAV delivery scheme proposed in Figure 6.

First, customers near the distribution center will be delivered to directly by UAVs, and these customer points will be removed in subsequent calculations (Figure 6a). Then, the remaining customer points are divided into five clusters and the positions of the corresponding vehicle stopping points are determined (Figure 6b). Following this, planning the delivery routes of vehicles and UAVs is carried out to determine the total time to serve all customers (Figure 6c). Finally, we can see the impact of the change in the number of vehicle stopping points K on the total service time (Figure 6d). When K=5, the inflection point appears and the overall optimal scheme can be determined. Figure 6e shows the results using vehicle delivery alone, which contrasts with the scheme proposed in this study.

### 5.2. Simulation Results

Fifteen cases were simulated and their results are shown in Table 3. Among them, some parameters need to be explained. The initial K value refers to the minimum number of clusters that meet the constraint conditions output by the algorithm for the first time, which produces the initialization result. The optimized K value refers to the optimal K value after increasing the number of clusters and calculating, which produces the optimized result. $Init\;T^{\prime }$, $Init\;T_{travel}$, and Init T refer to the waiting time of the vehicle at all stopping points, the vehicle running time, and the total time to serve all customers, respectively, at the initial K value. $T^{\prime }$, $T_{travel}$, and T refer to the results under the optimized K value. Then, the optimization ratio $R_{T}$ reflects the degree of optimization before and after optimization. $T_{truck}$ is the time required to complete the delivery using the vehicle alone under the same calculation example conditions. The efficiency improvement rate is $R_{effect}$, which reflects the improvement rate of the timeliness of the scheme proposed in this paper compared with the distribution scheme using vehicles alone.

**Figure 7:**
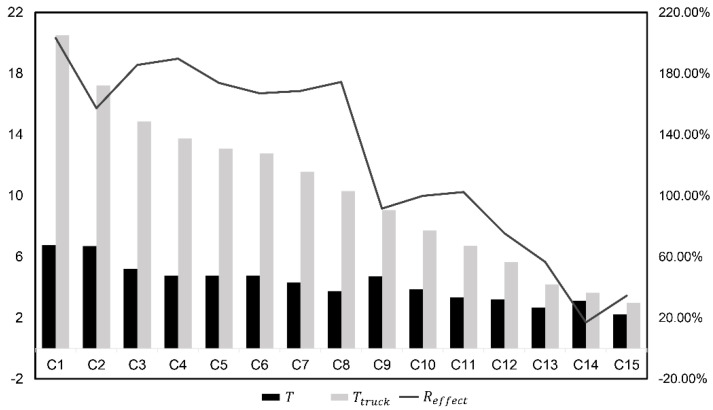
Efficiency of the algorithm.

Through the simulation results in Table 3, the vehicle-assisted UAV delivery scheme proposed in this paper is suitable for various examples of different scales, and relatively optimal results can be obtained. Judging from the initialization results and the results after optimizing the K value, the initialization results of most calculation examples were the optimal results. When the K value increased, it was often accompanied by a decrease in $T^{\prime }$ and an increase in $T_{travel}$. In addition, we found that when the scale of the calculation example was enlarged, it was easier to increase the cluster number K to significantly improve the overall efficiency such as the calculation examples C1, C2, C9, etc. On the other hand, through the analysis results in Figure 7, it can be concluded that compared with the traditional independent vehicle delivery, the proposed scheme had a significant improvement in efficiency. In the 15 cases, the efficiency improvement rate $R_{effect}$ ranged from 17% to 203.35%, and the average $R_{effect}$ was 126.5%. When the sample size was larger, the advantages of the proposed scheme were more obvious.

## 6. Conclusions

This study focused on the problem of vehicle-assisted UAV delivery in instant delivery scenarios. In response to a new problem where a vehicle is equipped with multiple UAVs and the UAVs serve multiple customers in a single take-off, a solution was proposed, taking into account the impact of changes in the payload of the UAV during the delivery process on energy consumption. First, a UAV energy-consumption model was established to constrain the energy consumption in the UAV-delivery process. Then, an UAV task-allocation model and a vehicle-path planning model were established to determine the UAV task allocation and path as well as the vehicle running path to obtain the minimum total service time. Finally, a hybrid heuristic algorithm was proposed to solve the problem, and it was verified by multi-scale calculation examples and comparative experiments. The research results showed that the solution to the problem of vehicle-assisted UAV delivery proposed in this paper can optimize the location of vehicle stopping points and the delivery routes of UAVs and vehicles and can help related companies that deal with instant delivery improve their operational efficiency.

However, there is still room for improvement and there are application challenges in this study such as for the estimation of UAV energy consumption. The assumptions we made are too strict, and the effects of wind and temperature on UAV energy consumption should be taken into account to fit a more accurate formula for estimating the energy consumption of UAVs while integrating low-cost sensors to cope with the unstructured and changing environment of UAV delivery.

## Figures and Tables

**Table 1:** Model notations.

Notation Type		Notation Description
Sets	C={0,1,2,…,n}	The set of all customer points, where 0 is the starting point of the UAVs
	$U=\{u_{1},u_{2},\ldots \}$	The set of all UAVs
	S={1,2,⋯s}	The set of vehicle stopping points
	K={0,1,2,⋯k}	The set of vehicle stopping points and a distribution center
	R={1,2,⋯r}	The set of service routes for each take-off of the UAVs
Parameters	$D_{c}$	The distribution center
	$t_{s}$	The service time of a single customer
	W	The maximum load of vehicle
	$V_{truck}$	The average speed of vehicle
	$W_{H}$	The maximum payload of the UAV
	$w_{d}$	The weight of the UAV
	P	The maximum flying power of UAV
	$E_{max}$	The total energy of the UAV
	$D_{max}$	The maximum flight radius of UAV under full load
	$V_{drone}$	The UAV flying speed at maximum power
	$w_{i}$	The weight of the package that should be delivered to customer i, i∈U
	$G_{i}$	The payload when the UAV leaves the customer (or stopping point) i, i∈C∪K
	$E_{ij}$	The energy consumed of the UAV leaves customer (or stopping point) i to customer (or stopping point) j, i,j∈C∪K
	$d_{ij}$	The distance of the UAV leaving customer (or stopping point) i to customer (or stopping point) j, i,j∈C∪K
	$t_{ij}$	The flight time of the UAV leaves customer (or stopping point) i to customer (or stopping point) j, i,j∈C∪K
	$v_{ij}$	The flight speed of the UAV leaves customer (or stopping point) i to customer (or stopping point) j, i,j∈C∪K
	$p_{ij}$	The distance between the vehicle stopping point i and j, i,j∈K
	$T_{u}$	The total service time of UAV u in cluster, u∈U
	$T_{s}^{\prime }$	The waiting time of the vehicle at the stopping point s
	$T^{\prime }$	The waiting time of vehicle at all stopping points
	$T_{travel}$	The vehicle running time
	T	The total time to serve all customers
Decision variables	$x_{ur}$(=1)	Binary$.\;x_{ur}$ Equation (1) if UAV u service line r in clusters
	$x_{rij}$(=1)	Binary$.\;x_{rij}$ Equation (1) if on route r the UAV travels from customer (or stopping point) i to customer (or stopping point) j
	$k_{ij}$(=1)	Binary$.\;k_{ij}$ Equation (1) if vehicle from stopping point i to stopping point j

**Table 2:** Simulation parameters and values.

Parameter	Value
UAV weight	$w_{d}\;$= 9 kg
UAV maximum load	$G_{i}\;$= 6 kg
UAV operating speed	$V_{drone}\;$= 45 km/h
UAV maximum flight power	P = 1.316 kW
Lift ratio	γ = 3
Conversion efficiency of the engine	η = 0.5
The total energy of the UAV	$E_{max}\;$= 0.31 kW·h
The energy loss of the UAV battery	e = 0.1 kW
The service time of a single customer	$t_{s}\;$= 0.05 h
The average speed of vehicle	$V_{truck\;}$= 35 km/h

**Table 3:** Simulation results of the example.

Example	Size	Initial K	Initial Result			Optimized K	Optimized Result			$R_{T}$	$T_{truck}$	$R_{effect}$
			Init T′	$Init\;T_{travel}$	Init T		$T^{\prime }$	$T_{travel}$	T			
C1	250	4	5.6458	1.1921	6.8379	5	5.5284	1.2324	6.7608	1.13%	20.5089	203.35%
C2	234	4	5.596	1.1543	6.7503	6	5.427	1.2708	6.6978	0.78%	17.234	157.31%
C3	218	3	4.41277	0.94203	5.3548	4	4.0158	1.1846	5.2004	2.88%	14.8541	185.63%
C4	202	5	3.6042	1.1446	4.7488	5	3.6042	1.1446	4.7488	0%	13.7586	189.73%
C5	186	4	3.6349	1.1378	4.7727	4	3.6349	1.1378	4.7727	0%	13.0729	173.91%
C6	170	4	3.6242	1.1828	4.807	5	3.4944	1.2881	4.7825	0.51%	12.7721	167.06%
C7	154	4	3.1998	1.1007	4.3005	4	3.1998	1.1007	4.3005	0%	11.552	168.62%
C8	138	3	2.84078	0.92892	3.7697	4	2.7467	1.0072	3.7539	0.42%	10.3038	174.48%
C9	122	5	3.6877	1.1703	4.858	6	3.3046	1.4208	4.7254	2.73%	9.0531	91.58%
C10	106	5	2.6696	1.193	3.8626	5	2.6696	1.193	3.8626	0%	7.7213	99.90%
C11	90	3	2.0828	1.245	3.3278	3	2.0828	1.245	3.3278	0%	6.7366	102.43%
C12	74	4	1.9551	1.2605	3.2156	4	1.9551	1.2605	3.2156	0%	5.64	75.39%
C13	58	3	1.6376	1.0418	2.6794	3	1.6376	1.0418	2.6794	0%	4.1955	56.58%
C14	42	4	1.9124	1.1892	3.1016	4	1.9124	1.1892	3.1016	0%	3.6304	17.05%
C15	20	2	1.4005	0.8231	2.2236	2	1.4005	0.8231	2.2236	0%	2.9905	34.49%

The optimization ratio $R_{T}=\frac{Init\;T-T}{Init}T*100\%$ the efficiency improvement rate $R_{effect}=\frac{T_{truck}-T}{T}*100\%$.

## Data Availability

The data presented in this study can be requested from the authors.
